# A Narrative Review of Retinopathy in Diabetic Patients

**DOI:** 10.7759/cureus.52308

**Published:** 2024-01-15

**Authors:** Somya Gupta, Archana R Thool

**Affiliations:** 1 Department of Ophthalmology, Jawaharlal Nehru Medical College, Datta Meghe Institute of Higher Education and Research, Wardha, IND

**Keywords:** hypertension, diabetes mellitus, blindness, photocoagulation, diabetic retinopathy

## Abstract

Patients with diabetes may be at risk of ocular diseases, like retinopathy due to diabetes and oedema of the eye. Patients with retinopathy due to diabetes experience constant injury to the retina and the posterior end of the eye, which is light-sensitive. It is a prominent complication faced by diabetics that threatens a patient's vision. Diabetes can inhibit the body's potential to ingest and maintain blood glycemic levels, resulting in several health problems. Excessive glucose in the blood can affect the eyes and other organs of the body. Diabetes has an effect on the blood supply system of the retina over a prolonged period of time. Diabetes-related retinopathy can lead to blindness as fluid can flow into the macula, which is essential for maintaining a clear visual field. The macula, despite its small size, is the region that enables us to comprehend colours and fine peculiarities well. The fluid swells the macula, leading to an impaired visual field. The weak, irregular blood vessels formed during neovascularization can potentially haemorrhage into the posterior end of the eye, obstructing the visual field. Blood vessels of the eye leak blood and other fluids, causing retinal tissue enlargement and eyesight clouding. Typically, the illness affects both eyes. Diabetes retinopathy is more likely to develop as a person's diabetes progresses. If untreated, retinopathy due to diabetes can result in blindness.

## Introduction and background

Today's society has a large number of cases of diabetes mellitus (DM). As per the WHO, diabetics in number will increase to sixty million by the next ten years [1]. Diabetes is one of the world's most widespread metabolic illnesses, characterised by faulty insulin release. In contrast, type 2 diabetes is distinguished by an increased insulin insufficiency and a comparable shortfall in insulin activation [2]. Increased glucose levels have proven to be a primary contributor to a number of complications. Diabetes comprises several overlapping and interconnecting pathways, which can lead to potential ocular manifestations such as retinopathy due to diabetes and oedema of the eye [3]. Diabetic retinopathy is predominantly a frequently caused consequence of insulin insufficiency and one of the most definite reasons for diminishing vision globally.

Retinopathy may stay steady or advance to diabetic oedema or proliferative diabetic retinopathy, both of which are significant contributors to profound vision loss in employed personnel, particularly in developed regions. Non-proliferative retinopathy differs from proliferative retinopathy in its morphological characteristic of neovascularization. Technological advancements have increased diagnostic accuracy and diabetic patients' access to specialised care. Treatment techniques have evolved over the previous three decades to include early surgical procedures and pharmacotherapies in addition to laser photocoagulation. Retinopathy due to diabetes is microangiopathy of the eye, which is characteristic in patients with prolonged diabetes. It is one of the most prevalent vascular diseases of the retina. After fifteen years of suffering from the disease, three out of every four diabetics suffer from retinopathy [4]. In the year 2020, retinopathy due to diabetes was the fifth most prevalent reason for premature vision loss and the fifth most pervasive cause of mild to severe vision loss in people aged 50. Diabetic retinopathy (DR) is strongly linked to a heightened risk of a vascular accident like the infarction of the heart and brain in the future [5].

## Review

Methodology

**Figure 1:**
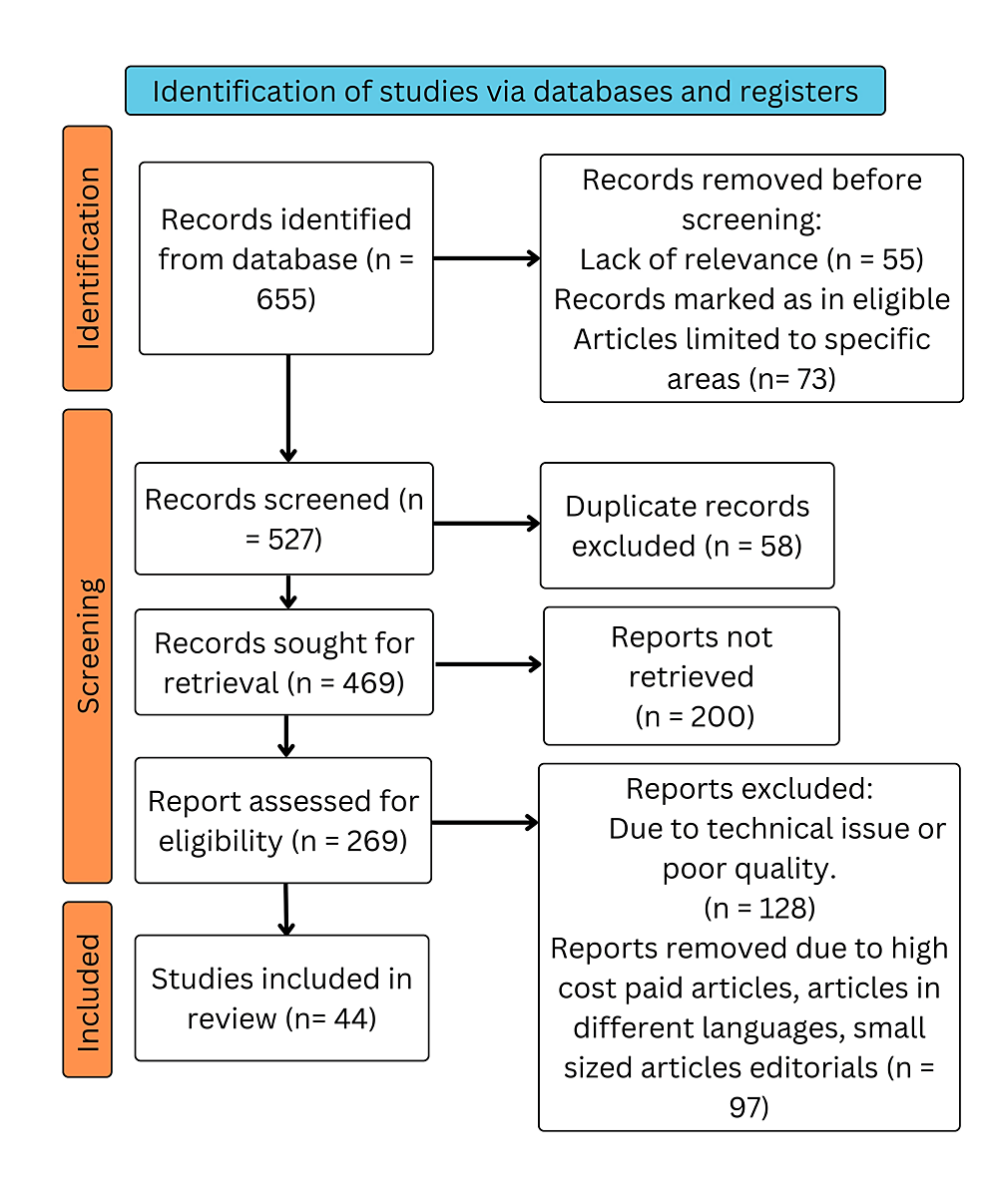
PRISMA methodology PRISMA: Preferred Reporting Items for Systematic Reviews and Meta-Analyses

We performed a comprehensive search in the electronic databases PubMed, MEDLINE (Medical Literature Analysis and Retrieval System Online), Embase (Excerpta Medica Database), Google Scholar, and ResearchGate, and a search of the English-language literature was done. It was also the subject of a different search. The query terms were "Pathophysiology", "Diabetic retinopathy" OR "Retinopathy due to diabetes", "risk factors" OR "progression", "Medical management of diabetic retinopathy" OR "Surgical intervention in diabetic retinopathy", and "Clinical manifestation of diabetic retinopathy" OR "Prevention and screening". The articles in this review meet the following requirements: studies conducted exclusively on pathophysiology and risk factors of DR, new treatment interventions and prevention of the disease, and studies conducted in English. Figure 1 highlights the Preferred Reporting Items for Systematic Reviews and Meta-Analyses (PRISMA) method used in the research methodology.

Classification of DR

Retinopathy with diabetes is classified into non-proliferative retinopathy and proliferative retinopathy [6]. Persistent intraretinal microvascular modifications are characteristic in patients with non-proliferative retinopathy. Proliferative retinopathy is distinguished by neovascularization in the retina or the optic disc [7]. A recent systematic review found that in patients with diabetes aged 20 to 79, the overall prevalence of retinopathy due to diabetes is around 34.6% and 7.0% for proliferative DR (PDR). DR progresses from mild to moderate and severe non-proliferative DR (NPDR). It is characterized by persistent ocular microvascular rupture and ocular ischemia. PDR is marked by neovascularization on the optic disc and retina. Neovascularization is usually followed by producing fibrous tissue, which causes vitreous bleeding and retinal detachments. Improper treatment might lead to the progression of PDR to an involutional dormant state. The advancement of visual defects is determined by the extent of harm to essential structures at the site of the retina [8].

Risk factors

Time Scale of Diabetes 

The frequency and graveness of retinopathy with diabetes are directly related to the stretch of DM in a patient [9].

Glycemic Control

Glycemic management and the advancement of retinopathy due to diabetes have an indirect link. The Diabetes Control and Complications Trial (DCCT) and the Early Treatment of Diabetic Retinopathy Study (ETDRS) presented that rigorous medical attention decreases the likelihood of advancement of retinopathy in diabetics. Reduction in glycosylated haemoglobin levels has been linked to a substantial decline in the progression of retinopathy [10].

Nephropathy

High levels of gross proteinuria have been linked to a 95% increase in the probability of developing oedema in type 1 diabetics as stated in The Wisconsin Epidemiologic Study of DR (WESDR). PDR was considerably more common in people with chronic microalbuminuria [11].

Genetics

Patients with HLA DR4 along with absent HLA DR3 levels are more likely to progress to proliferative retinopathy as mentioned in WESDR. The DCCT data also revealed a genetic propensity to diabetes. However, environmental and genetic factors are thought to participate in the emergence of retinopathy due to diabetes [12].

Serum Lipid Level 

Increased blood cholesterol is linked with a higher risk of retinal sclerosis. WESDR and ETDRS found a link between lipids present in the serum and the risk of exudates in the retina in type 2 diabetes. Gupta et al. recently concluded that utilising atorvastatin as an adjuvant not only reduced oedema but also the grievousness of solid exudates and subfoveal lipids in individuals with diabetes [13].

Anaemia

Low baseline hematocrit levels were established as a primary indicator for the emergence of high-risk proliferative retinopathy in diabetics and severe blindness in the study of ETDRS. It was concluded that patients with haemoglobin levels less than 12 g/dl had an increased risk of retinopathy [14].

Socioeconomic Status

Socioeconomic status did not appear to correlate with a higher probability of degradation of the retinopathy. If the degree of insulin is under scrutiny, social variables have a negligible impact on these complications of diabetes [15].

Gestation Period

Pregnant women with type 1 diabetes are twice as susceptible as non-pregnant women to acquiring PDR. Young moms should ideally be tested for retinopathy before becoming pregnant [16]. The cause of DR acceleration could be a simple reflection of diabetes's extended duration [17].

Pathophysiology

Mild DR is distinguished by the hyperpermeability of blood vessels. Progression to moderate, mild and severe NPDR is characterised by persistent retinal capillary leakage or atrophy, leading to retinal infarction. PDR is distinguished by neovascularization examined by fundoscopy of the optic disc. These new vascular growths are typically accompanied by the production of fibrous tissue, which causes vitreous haemorrhage and tractional retinal detachments (TRDs). PDR will invariably progress to an advanced diabetic eye disease with TRD. The deteriorating visual acuity (VA) level is determined by the extent of essential structures damaged at that moment. Laser pan-retinal photocoagulation produces the dormant state which inhibits further progression of the disease [18].

DR: Genomic and Inorganic Processes

**Figure 2:**
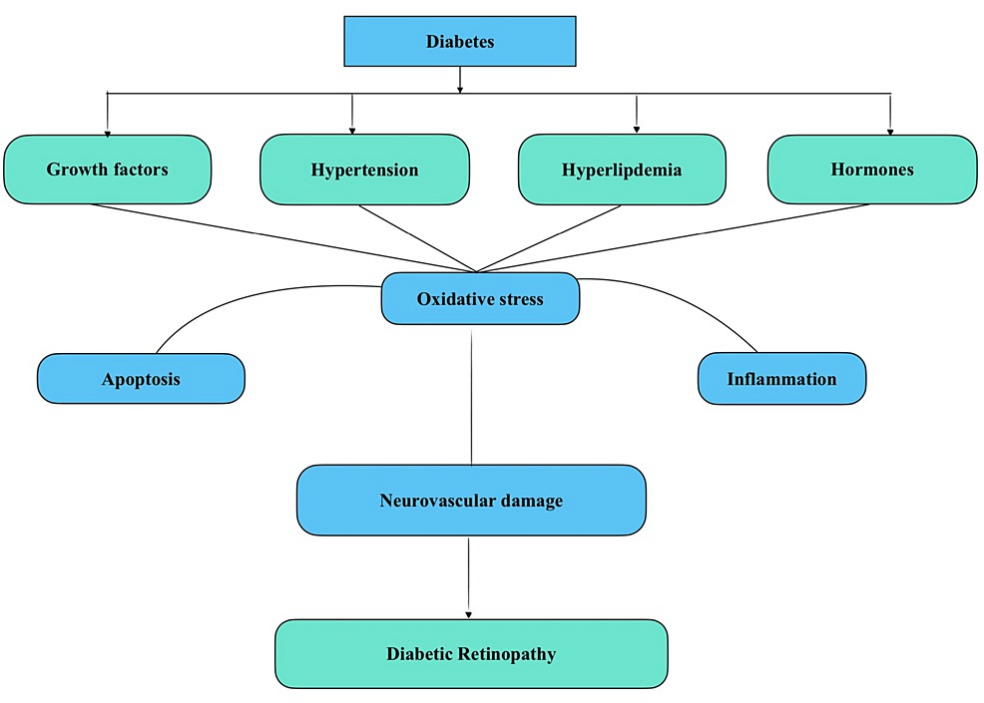
Pathogenesis of diabetic retinopathy Image Credit: Author Somya Gupta

Several analyses have been conducted to characterise the method involved in both the onset and progression of retinopathy due to diabetes; nevertheless, no mechanism can be considered proven. Diabetes is characterised by raised blood glucose levels, insulin resistance, a slight or absolute lack of insulin activity, and the development of diabetes-specific pathology in the retina [19]. Retinopathy due to diabetes is one of the most prominent reasons for visual impairment worldwide. Pericyte loss, basement membrane thickening, microaneurysms, neovascularization, and blood-retinal barrier disruption are the primary signs of retinopathy due to diabetes [20]. Enhanced glucose flux through the hexosamine pathway led to activation of protein kinase C and expanded glycation end-product, which all have been linked with diabetic retinopathy (Figure 2) [21]. 

Clinical manifestation of DR 

Non-proliferative retinopathy due to diabetes is characterised by microaneurysms, one of the first medically apparent pathologies of retinopathy due to diabetes. Dot and blot bleeding are found in the inner layer of the retina, specifically in the intermediate layers. On the contrary, hard exudates and cotton wool spots are present in the retinal layer between the inner plexiform and inner nuclear layers. Vascular alterations such as beading, looping, and dilation of vein segmentation are present in the nerve fibre layer. Infarction of the nerve fibre is caused due to blockage of the nerve fibres of the retina. Intraretinal microvascular abnormalities (IRMA) are dilated capillaries and are typically encountered next to capillary closure zones, distinguished by oedema of the retina [22].

Screening of retinopathy due to diabetes 

Ophthalmoscopy

The most prevalent procedure for screening for DR is ophthalmoscopy. When conducted by an ophthalmologist, direct and indirect ophthalmoscopy has a high specificity but low sensitivity level, especially for early retinopathy, compared to a 7-field stereo photographic examination [23].

Imaging of the Fundus

Digital imaging enables photographic documentation of the fundus to be more convenient and readily available. It can be utilized to acquire images of the fundus through pupils that are not dilated. A mydriatic agent is usually required in elderly people for easy investigation. Single-field fundus photographic imagery with skilled interpretation can be used as a diagnosing method to detect retinopathy due to diabetes [24]. Fundus imagery has made it possible to do an easy screening of various retinal diseases under the supervision of fewer trained healthcare professionals as compared to before. In broader terms, a fundus photo is acquired with a fundus camera, a microscope with minimal power that can record retinal components such as the optic disc, cup, macula and posterior pole. Fundus imagery has been identified as an accessible and effective approach that enables ophthalmologists to promptly analyze and assess photos acquired from diverse locations, including rural and metropolitan regions. Subsequently, it can facilitate appropriate medical recommendations and therapies. It is to be noted that fundus angiography is used to diagnose retinopathy due to diabetes using three different image modalities: (i) red, green, and blue (RGB) imaging modalities, (ii) red-free channel, and (iii) fluorescent imagery [25].

Performance Measure

The benefit of adopting computer-aided diagnosis (CAD) for diabetes-related retinopathy is the ability to build a real-time diagnostic system that allows patients and healthcare providers to respond quickly. To test and confirm the usefulness of these CAD procedures against the established manual assessment offered by qualified experts, multiple statistical coefficients such as hypersensitivity, particularity, accuracy, predictability, F1-score, and area under the curve are used. This comparison aids in the establishment of an objective link between various techniques [26]. 

Management of retinopathy due to diabetes 

Laser Photocoagulation

In clinically significant macular oedema (CSME) and retinopathy with high-risk factors, laser photocoagulation is advised as the basic line of treatment in hand [27]. Pregnancy, nephropathy, heart failure, heart disease, cataract surgery, Yag capsulotomy, and unsupervised blood glucose levels are high-risk factors known to worsen retinopathy at a faster rate. Insulin initiation in non-insulin-dependent DM (NIDDM) patients with constantly administrated blood glucose levels and poor patient monitoring worsen the condition and may prompt prognosis in severe non-proliferative retinopathy and early proliferative retinopathy [28]. When new blood vessels continue to form or some areas do not receive complete treatment following panretinal photocoagulation, further treatment is administered. Burns are administered to previously treated laser markings that have been skipped, particularly at the centre of the optic disc and macula. Furthermore, treatment is directed at locations where new blood vessels are forming [29].

Pars Plana Surgery

The pars plana vitrectomy (PPV) procedure is routinely used to treat different forms of retinopathy. Vitreous bleeding, tractional retinal detachment, and mixed tractional-rhegmatogenous detachment are among the most common symptoms [30]. Macular oedema with thickened and tense posterior hyaloid, macular heterotopia, epiretinal membrane, severe macular haemorrhage, neovascular glaucoma with clouded media, and ghost cell glaucoma are less commonly encountered findings [31].

Pharmacotherapy

Pharmacological medications can influence the metabolic process at various stages, mitigating complications arising from diabetes, including retinopathy, dermatopathy, neuropathy, and nephropathy. Many diabetes-related issues, such as eye oedema and the formation of new blood vessels, stem from the release of developmental factors triggered by retinal infarction resulting from alterations in the configuration and molecular structure of the microvasculature [32]. In reaction to hypoxia damage to pigment epithelial cells, pericytes, and endothelial cells of the retina release vascular endothelial growth factor (VEGF). VEGF promotes inflammation by increasing the production of intracellular adhesion molecule-1 (ICAM-1) and conformity of white blood cells [33].

Measures to prevent retinopathy due to diabetes 

Today's clinical approach to the prevention and therapy of DR is based on regulating sugar and blood pressure levels rather than on DR-specific pharmaceutical medications [34]. The results of a meta-analysis and randomised review of scientific studies indicate that strict control of glucose levels reduces the incidence of DR by 20 per cent [35]. Managing high blood pressure can also effectively minimize the risk of DR development [36]. Medical care of high levels of lipids in the body may effectively ease DR. Patients with type 2 diabetes with increased cholesterol levels who took medication had a reduced rate of DR advancement in four years [37].

Primary Prevention

The first step for diabetes is to make lifestyle changes. Modifications before taking medications, such as a nutritious diet and a physical activity program, are the foundation of diabetic management. The primary goals include maintaining good nutrition, a balanced weight, normal cholesterol levels, and effective insulin management, which are essential considerations in type 2 diabetes. The required fundamental lifestyle changes in type 2 diabetes include routine physical activity, intelligent eating choices, and weight loss [38]. 

Secondary Prevention

Present approaches to therapy and precise analytical criteria for DR treatment, management, monitoring, and understanding have significantly lowered the danger of blindness from retinopathy due to diabetes and macular oedema. Laser photocoagulation and vitrectomy have enhanced their standard of life and averted sight loss in DR patients [39].

Discussion

Retinopathy due to diabetes is a common complication of poorly treated sugar diabetes, which might cause severe blindness and blurred vision in the general population. Retinopathy now affects roughly one hundred fifty million individuals globally, with the WHO projecting that the number will double by 2025 [40]. Patients with diabetes face many potentially lethal secondary symptoms, including macrovascular-related stroke, cardiomyopathy, coronary artery syndrome, microvascular-related retinal degeneration, neuropathy, and nephropathy. The majority of frequent microvascular consequences of diabetes is retinopathy due to diabetes [41]. DR, relative to other vascular sequelae, is linked with neovascularization, whereas insulin-dependent coronary artery disease and nephropathy due to diabetes are characterized by reduced revascularization [42]. Some research implies that the frequency of diabetes-related blindness has decreased in recent years in the United States; this is mainly owing to advances in systemic management [43]. Retinopathy due to diabetes is an increasing worldwide problem. DR now affects around a hundred million individuals worldwide and is anticipated to turn into a severe health concern, with figures indicating that retinopathy-related blindness increased by twenty-seven percent in the year 2010 [44].

Summary table 

All the studies included in this review are summarised in Table 1.

**Table 1:** Summary of the studies included in the review VEGF: Vascular endothelial growth factor; ACCORD: Action to Control Cardiovascular Risk in Diabetes

Author	Year	Findings
Wild et al. [1]	2004	Worldwide consequences faced due to diabetes with the estimates for the present, that is, for the 2000s and for the near future.
Pirart et al. [2]	1978	Diabetes mellitus and its worsening complications: a 4,400-patient potential analysis.
Nguyen et al. [3]	2010	Findings of a two-year investigation into ranibizumab for macula oedema in diabetic patients.
Klein et al. [4]	1989	Diabetes Retinopathy Epidemiologic Study in Wisconsin, United States. When the age of diagnosis is 30 years or older, the occurrence and development of diabetic retinopathy is four years.
[5]	2021	Determinants for visually impaired people in 2020 and patterns over 30 years, as well as the incidence of preventive blindness, are all linked to Vision 2020: The Right to Sight, a Global Burden of Disease study.
Modjtahedi et al. [6]	2021	Diabetic retinopathy of grave severity, present with the risk of subsequent cerebrovascular pathology, and mortality.
Wilkinson et al. [7]	2003	Clinical research indicating the severity of diabetic retinopathy and oedema on a comparative level.
Amoaku et al. [8]	2020	Study groups in the United Kingdom summarising the pathogenesis of retinopathy and oedema due to diabetes among people.
Yau et al. [9]	2012	The global incidence and indicators for the pathogenesis of retinopathy due to diabetes are summarised in this study.
Nathan et al. [10]	1993	The influence of extensive therapy on the advancement of chronically presenting symptoms in insulin-dependent patients.
Chaturvedi et al. [11]	2001	Microalbuminuria is discussed as a risk factor affecting the glycemic threshold in type 1 diabetic patients.
[12]	1997	The research group complied the chronic effect of diabetes in one’s body leading to a variable amount of complications.
Gupta et al. [13]	2004	Drug atorvastatin as a substitute is used in the therapy regimen of macular oedema due to its lipid-lowering properties.
Davis et al. [14]	1998	The Preliminary Management of Diabetic Retinopathy Study Report emphasised the high-risk factors of the disease.
Klein et al. [15]	1994	The relationship between social and economic factors involved in the prevalence of proliferative diabetic retinopathy and visual loss is presented here.
Klein et al. [16]	1990	The relationship between pregnancy and the development of diabetic retinopathy is summarised here.
Horvat et al. [17]	1980	A 12-year prospective survey focusing on the good and ill effects of pregnancy on the retinopathy of the eye.
[18]	1978	The report of Diabetic Retinopathy Study focusing on photocoagulation as a non-invasive treatment regimen for retinopathy due to diabetes.
Safi et al. [19]	2014	Therefore, in the near future, pharmacologic management approaches may involve multiple drugs targeting various mechanisms aggressively.
Fong et al. [20]	2003	Management modalities that can serve as prophylaxis for diabetic retinopathy, aiming to prevent blindness in a significant portion of the population with diabetes. The authors came to the conclusion that glycemic and blood pressure control can retard the development of retinopathy in the eye.
Brownlee M et al. [21]	2001	Increased superoxide generation by the mitochondrial chain focusing on the electron transport system. This integrated approach proposes a new foundation for further studies in favour of the mentioned disease.
[22]	1991	Here the treatments providing the chance of visual improvement, decreasing the cases of recurrent macular oedema, and resulting in only minor ocular manifestation were discussed.
Lin et al. [23]	2002	Sensitivity of digital fundus photography along with specificity of diabetic retinopathy with the help of presented monochromatic images.
Williams et al. [24]	2004	A digital photograph of the optic disc and macula was used for the precise diagnosis of diabetic retinopathy in individuals as it was a wide-field image in nature and highly sensitive.
Qureshi et al. [25]	2005	Effective digital fundal images were utilized for the diagnosis of diabetic retinopathy.
Besenczi et al. [26]	2016	One can argue that thorough prospective and descriptive analysis of medical records, including retinal pictures, has the potential to successfully enhance the prognosis of the disease.
Besirli et al. [28]	2009	Ischemia of the numerous layers of the retina due to occlusion of the retinal capillary present in the fungus causes neovascularization of the retina in proliferative diabetic retinopathy.
Singh et al. [29]	2008	The present investigation covers the scope of the problem in India, as well as traditional and ongoing initiatives to prevent potentially blinding consequences due to diabetes.
Vander et al. [30]	2000	Here the authors assert that the current clinical standards for surveillance of diabetic retinopathy are futile, as evidenced by low compliance with the recommendations.
Spraul et al. [31]	1997	Pars plana vitrectomy as a choice of treatment for chronic haemorrhage of the vitreous.
Aiello et al. [32]	1994	The involvement of VEGF in the progression of diabetic retinopathy and other retinal illnesses is summarised by the author.
Ishida et al. [33]	2003	The aim as assured by the author was to determine the relative efficacy of two main VEGF isoforms, VEGF120 and VEGF164, in causing leukocyte stasis (leukostasis) inside the retina.
Clark et al. [34]	1995	Though diabetes is often associated with premature disease of the eye, this review focused on retinopathy, nephropathy, and neuropathy. The pathogenesis of these diabetes consequences was discussed.
[35]	1998	The article focused on the comparative study of the control blood glucose one level along with sulphonylureas and its emphasis on the risk of further progression of the disease in patients.
[36]	1998	This study examined the results of control of blood pressure leading to complications in type 2 diabetes.
Chew et al. [37]	2010	The ACCORD Study looked into the impact of clinical treatments on the advancement of retinopathy in diabetics.
Gupta et al. [38]	2013	This article discussed the relationship between diabetic retinopathy and VEGF.
Kitada et al. [39]	2003	The article explored the translocation of glomerular p47phox and p67phox induced by protein kinase C activation and their role in oxidative stress in diabetic nephropathy.
King et al. [40]	1998	This publication provided the worldwide burden of diabetes from 1995 to 2025, which included incidence and projections.
Duh et al. [41]	1999	This article focused on the role of VEGF in diabetes, dealing with both its agonistic and antagonistic aspects.
Antonetti et al. [42]	2012	A comprehensive review of diabetic retinopathy; covered its various aspects.
Klein et al. [43]	2010	This publication discussed a long-term epidemiological perspective on vision in diabetics.
Leasher et al. [44]	2016	In this meta-analysis from 1990 to 2010, the global numbers projected the estimate of people who went blind or suffered from visual impairment as a result of diabetic retinopathy.

## Conclusions

In conclusion, this comprehensive review highlights the multifaceted nature of DR, encompassing its pathogenesis, risk factors, diagnostic methods, and evolving treatment strategies. Future research should focus on improving diagnostic procedures, developing specialised medicines, and developing preventive strategies targeted at minimising the global burden of diabetes-related comorbidities. Collaboration among researchers, clinicians, and public health initiatives will ultimately be essential in achieving better outcomes for individuals affected by diabetic retinopathy. Significant advances in diabetic retinopathy have been accomplished in recent years, altering and completely changing healthcare and educational agendas in the coming years.

This review is focused on the aetiology, risk factors, and pathogenesis of the ocular disease. Through this review, we tried to go through the significant events concerning managing and preventing the ocular disease of diabetic retinopathy. This article tried to foresee and predict some of the trends that are likely to have an impact over the years to come. While many new imaging, diagnostic, and treatment technologies can significantly enhance the medical consequences of diabetic retinopathy, these advancements must be applied equally to high- and low-resource settings worldwide. Significant advancements have been achieved in the field of diabetic retinopathy, and ongoing technological developments are continually enhancing the prognosis of the disease. Many ongoing advancements will significantly impact medical and research in the next decade. In this article, we examined some of the most recent advances in the area and speculated on how these advancements may continue to impact the population in 2030 and beyond. 
